# Efficiency improvement of InGaP/GaAs/Ge solar cells by hydrothermal-deposited ZnO nanotube structure

**DOI:** 10.1186/1556-276X-9-338

**Published:** 2014-07-05

**Authors:** Chen-Chen Chung, Binh Tinh Tran, Kung-Liang Lin, Yen-Teng Ho, Hung-Wei Yu, Nguyen-Hong Quan, Edward Yi Chang

**Affiliations:** 1 Department of Materials Science and Engineering, National Chiao Tung University, 1001 University Road, Hsinchu 300, Taiwan; 2 Industrial Technology Research Institute, Mechanical and Systems Research Labs, 195, Sec. 4, Chung Hsing Road, Chutung, Hsinchu 31040, Taiwan

**Keywords:** ZnO nanotube, Triple-junction, Hydrothermal

## Abstract

In this paper, a zinc oxide (ZnO) nanotube, fabricated by the hydrothermal growth method on triple-junction (T-J) solar cell devices to enhance efficiency, is investigated. Compared to those of bare T-J solar cells (without antireflection (AR) coating) and solar cells with Si_3_N_4_ AR coatings, the experimental results show that the T-J solar cells, which use a ZnO nanotube as an AR coating, have the lowest reflectance in the short wavelength spectrum. The ZnO nanotube has the lowest light reflection among all experimental samples, especially in the range of 350 to 500 nm from ultraviolet (UV) to visible light. It was found that a ZnO nanotube can enhance the conversion efficiency by 4.9%, compared with a conventional T-J solar cell. The Si_3_N_4_ AR coatings also enhance the conversion efficiency by 3.2%.The results show that a cell with ZnO nanotube coating could greatly improve solar cell performances.

## Background

In solar power technologies, the III-V solar cell is so far the commercial solar cell with the highest efficiency. It is expected that III-V solar cell will play an important role in the future high-efficiency and low-cost photovoltaic cell industry [1]. Triple-junction (T-J) solar cells composed of three subcells, namely,InGaP (top cell, band gap energy Eg = 1.9 eV), GaAs (middle cell, Eg = 1.42 eV), and Ge (bottom cell, Eg = 0.67 eV), are GaAs-based solar cells which achieved conversion efficiencies of over 40% and have been applied extensively to space and terrestrial use [2,3]. For high-performance multi-junction solar cells, the antireflection plays an important role because it can reduce about 30% of the light absorption due to the reflection between the interface of the air and top cell. The excellent antireflection (AR) performance benefits from the rough interfaces between air/zinc oxide (ZnO) nanotube layers and the ZnO nanotube/solar cell; the decreased nanotube densities provide the gradient of effective indices. The nanostructures have been applied to photovoltaic devices to reduce reflectance. The AR nanostructure is also described elsewhere [4-7]. Nanostructure arrays, like the subwavelength structures, exhibit very low specular or total reflectance compared to film layers. The low reflectance is due to a combination of AR coating and light tapping structures, demonstrating the nanostructure can potentially be applied a PV [8,9]. ZnO has been recognized as a very promising material for optoelectronic application in the UV region; so, there is an increasing interest in high-quality ZnO film. ZnO has a wide bandgap of 3.37 eV and has a large exciton binding energy (60 meV) at room temperature [10,11]. Compared with the planar thin-film devices, nanostructure devices are expected to have a greater response to light, especially for the spectrum from ultraviolet (UV) to green light in the solar spectrum [12,13], which can increase light absorption in the top cell for short wavelengths. For solar cells, ZnO thin film acts as a transparent conductive oxide (TCO) and AR layer (refractive index of 2.0). There is a great deal of information on fabricating one-dimensional (1D) ZnO nanotubes using chemical vapour deposition for high-quality transistor devices, which requires a high-temperature process, ranging from 400 to 1,050°C [14,15]. However, the high temperatures required for the CVD process degrade the characteristics of the solar cells. The ZnO nanotube with interest stemming from the facile synthesis with aligned and uniform ZnO nanotube arrays by using low-temperature (below 100°C) hydrothermal methods was also tried on the solar cells, without degrading the properties of the solar cells [16]. In this study, the growth of a ZnO nanotube on T-J solar cells via the hydrothermal growth method is investigated. The main motivation behind this study is the fact that nanostructures will act as a second ARC layer with an effective refractive index so that the refractive index of the total structure will perform as a double-layer AR coating layer. The optical and electrical properties ofthe III-V solar cells with the above-proposed double-layer AR coating in this study are measured and compared.

## Methods

**Figure 1:**
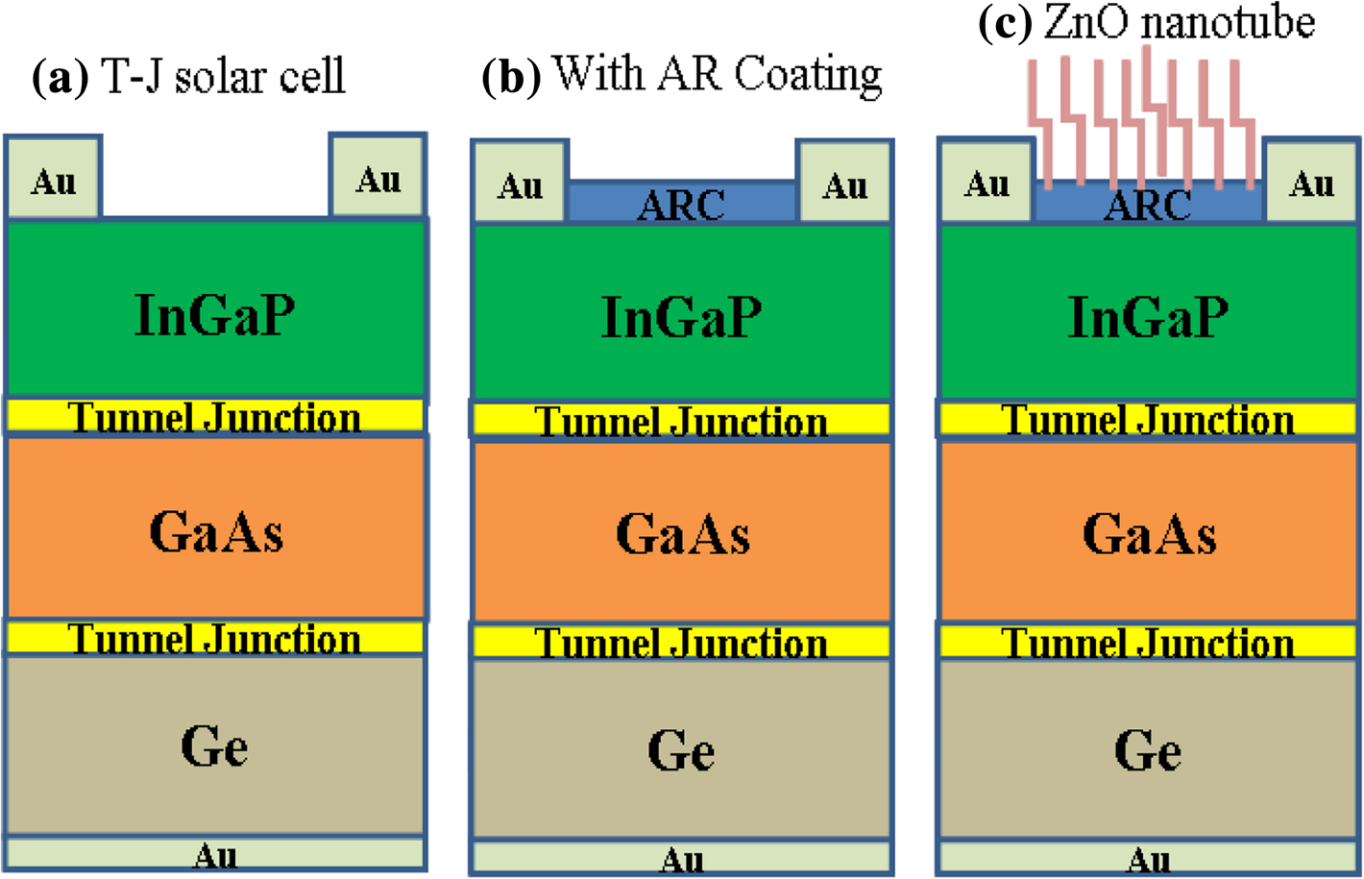
**Schematic of device processing step used in this study. (a)** Bare T-J solar cell. **(b)** With Si_3_N_4_ AR coating T-J solar cell. **(c)** ZnO nanotube T-J solar cell.

The epitaxial structure of the InGaP/GaAs/Ge T-J solar cells used in this study is shown in Figure 1. The structure was grown on p-type Ge substrates using a metal organic chemical vapor deposition system (MOCVD). During epitaxial growth, trimethylindium (TMIn), trimethylgallium (TMGa), arsine (AsH_3_), and phosphine (PH_3_) were used as source materials of In, Ga, As, and P, respectively, and silane (SiH_4_) and diethylzinc (DEZn) were used as the n-type and p-type doping sources, respectively. The epitaxial layers of the T-J solar cells were grown on a p-type Ge substrate at 650°C with a reactor pressure of 50 mbar [17]. After the epitaxial layers were grown, the wafers were cleaned using chemical solutions of trichloroethylene, acetone, methanol, and deionized water and dried by blowing N_2_ gas. A back electrode Ti (500 Å)/Pt (600 Å)/Au (2,500 Å) was then deposited immediately on the cleaned p-type Ge substrate using an electron-beam evaporator. Metal was annealed at 390°C for 3 min in an H_2_ ambient for ohmic contact formation. The front-side n-type contact was formed by deposition of Ni/Ge/Au/Ni/Au with a thickness of 60/500/1,000/400/2,500 Å. The 75-nm silicon nitride AR coating film was deposited using the plasma-enhanced chemical vapor deposition (PECVD) system on the solar cell device. The shadow loss due to the front contacts was 6.22%, and the total area of the solar cell was 4.4 × 4.4 mm^2^ with an illuminated active area of 0.125 cm^2^. After the device process was finished, a ZnO nanotube was grown using the hydrothermal method. The substrate was vertically positioned in a 60-mL mixture with 40 mL of zinc nitrate hexahydrate (Zn(NO_3_)_2_‧6H_2_O) (0.025 mol/L) and 10 mL of hexamethenamine (C_6_H_12_N_4_ (0.025 mol/L)). The substrate was then placed into a metal can with a capacity of 100 mL. The metal can was sealed and heated at 90°C making it easy to fabricate over a large area. Therefore, the ZnO nanotube fabrication technology has a potential which can be applied to the commercial process for the solar cell industry. The surface morphology of the ZnO nanotube was characterized by a field-emission scanning electron microscope (Hitachi S-4700I, Tokyo, Japan). The reflections of the samples were analyzed with an ultraviolet-visible (UV-VIS) spectrophotometer using an integrating sphere. For solar cell measurement, the current-voltage (I-V) characteristics of the samples were measured under a one sun AM1.5 (100 mW/cm^2^) solar simulator.

## Results and discussion

**Figure 2:**
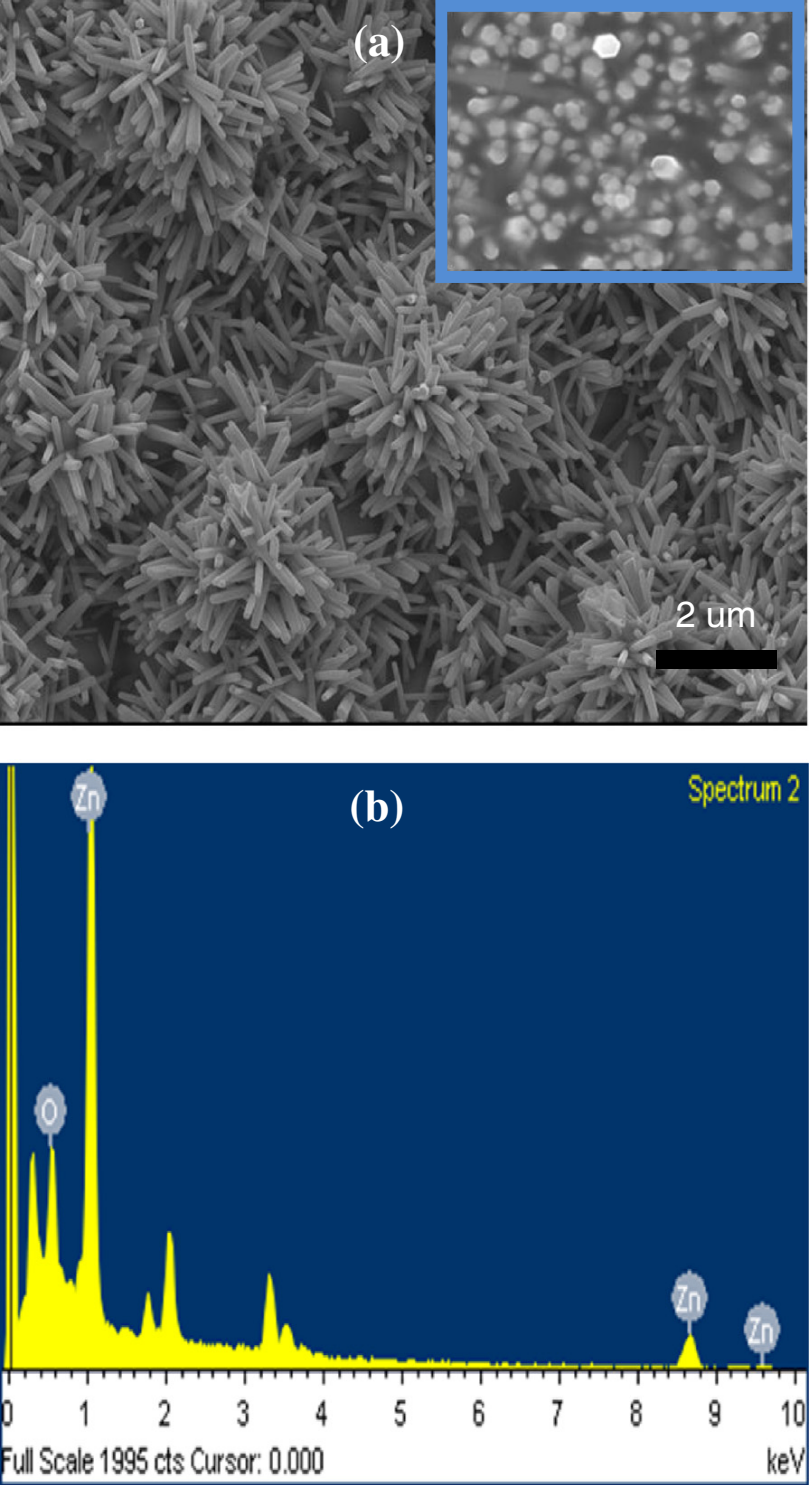
**SEM images and Energy dispersive spectrometer image of ZnO nanotubes. (a)** Plan-view SEM images of the ZnO nanotube structure. **(b)** Energy dispersive spectrometer (EDS) image of ZnO nanotube.

**Figure 3:**
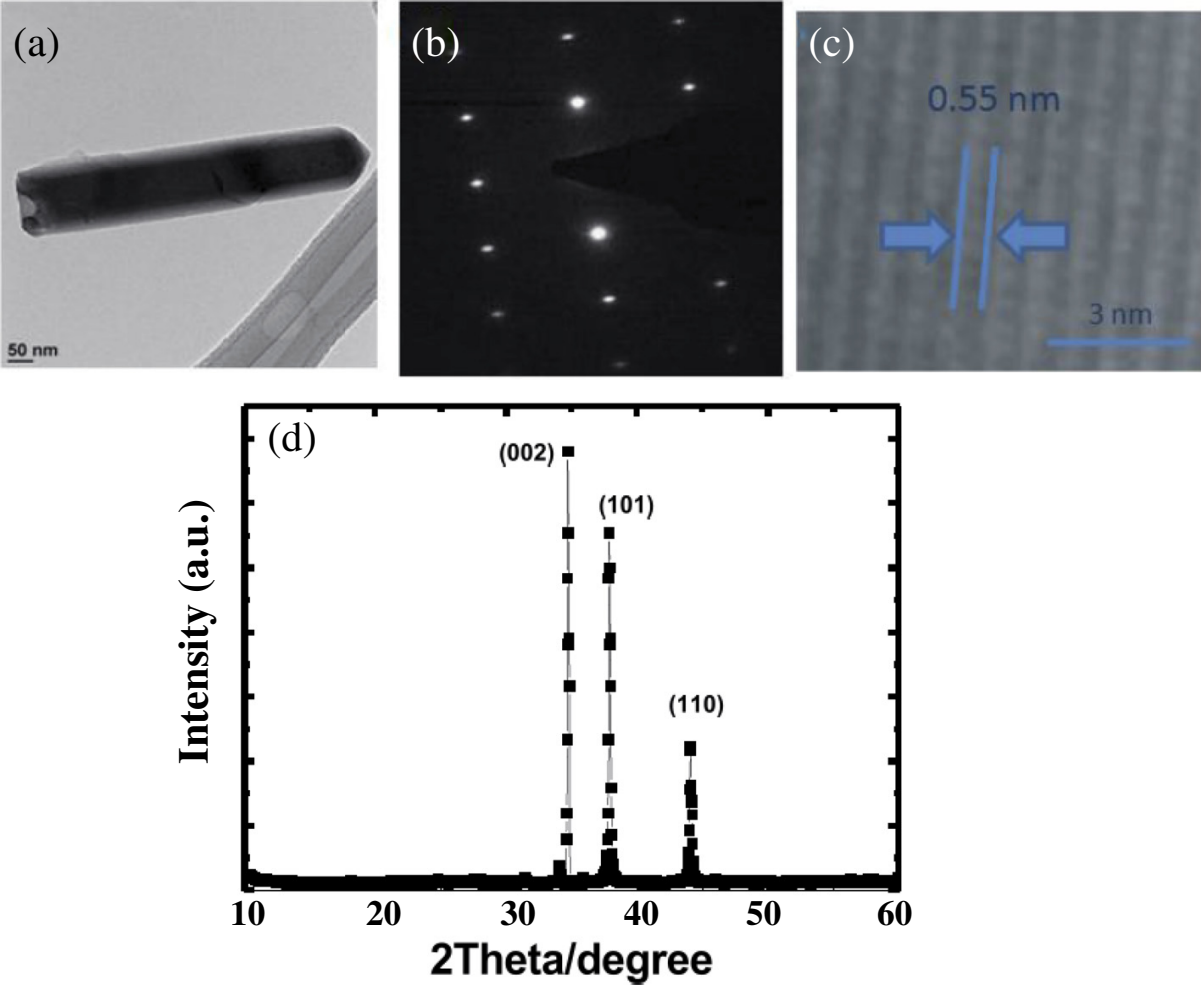
**TEM image, SAED, high-resolution TEM image, and X-ray diffraction pattern of ZnO nanotube. (a)** TEM image of ZnO nanotube, **(b)** the corresponding SAED of the ZnO nanotube, **(c)** a high-resolution TEM image of the ZnO nanotube, and **(d)** X-ray diffraction pattern of ZnO nanotube grown on solar cell.

Figure 2a shows the top view of SEM images of the ZnO nanotube structure. The hydrothermal growth method depends on the polarity of the ZnO crystalline structure, which allows for self-alignment into a wurtzite shape. The structure of the ZnO nanotube arrays varied with the different diameters of the nanotubes (80 to100 nm). Figure 2b shows the energy dispersive spectrometer (EDS) image of a ZnO nanotube. It shows clearly the Zn and O elements on the cell. In a solar cell, the high performance of antireflection coating (AR coating) determines the efficiency. An AR coating on the top with a broadband low-reflectance characteristic is crucial for most solar cells. TEM was used to further investigate the microstructure of the as-synthesized ZnO nanorod arrays. Figure 3a shows a bright field TEM image of a single ZnO nanotube. The diameter of the selected nanotube was uniform along the growth direction and was about 80 nm. The corresponding selected area electron diffraction (SAED) is shown in Figure 3b; it indicates that the nanotube grew along the [0001] direction, the fastest growth direction of ZnO. A high-resolution (HR) TEM image in Figure 3c shows the same result with the SAED pattern and indicates that the synthesized ZnO nanotube possessed a wurtzite single-crystal structure. Figure 3d shows an X-ray diffraction pattern of a ZnO nanotube grown on a T-J solar cell. A strong (002) diffraction peak and various (101), (110), and (002) peaks can be observed. These results indicate that (002) is the main growth plane, which is perpendicular to the c-axis, and that the ZnO nanotube grew preferentially along the c-axis.

**Figure 4:**
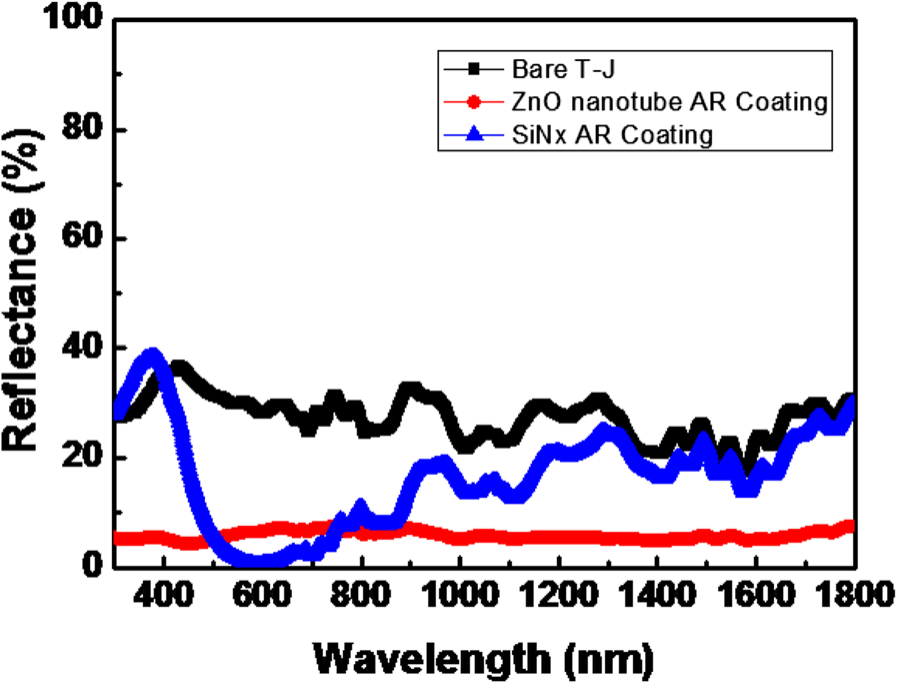
**Reflectance values of bare T-J solar cell and T-J solar cells with Si**_**3**_**N**_**4 **_**and ZnO nanotube coating, respectively.**

Figure 4 shows the reflectance values of a bare T-J solar cell and T-J solar cells with Si_3_N_4_ and ZnO nanotube coating, respectively. Since the ZnO nanotube can suppress light scattering at short wavelengths, the T-J solar cell with a ZnO nanotube has the lowest reflectance, especially in the wavelength range of UV to green. The weighted reflectance of the ZnO nanotube is approximately 5.7% for the wavelength range of 300 to 1,800 nm, which is still lower than that of a cell with Si_3_N_4_ which is approximately 18.1%. The cell with a ZnO nanotube shows a lower optical reflectance for wavelength from 300 to 1800 nm. Hence, the T-J with a ZnO nanotube exhibited superior antireflective properties for the wavelength range of interest. Low reflectivity in the UV to green light wavelength range gives promise for multi-junction solar cells if more junctions are requested especially for high-band gap subcells.

**Figure 5:**
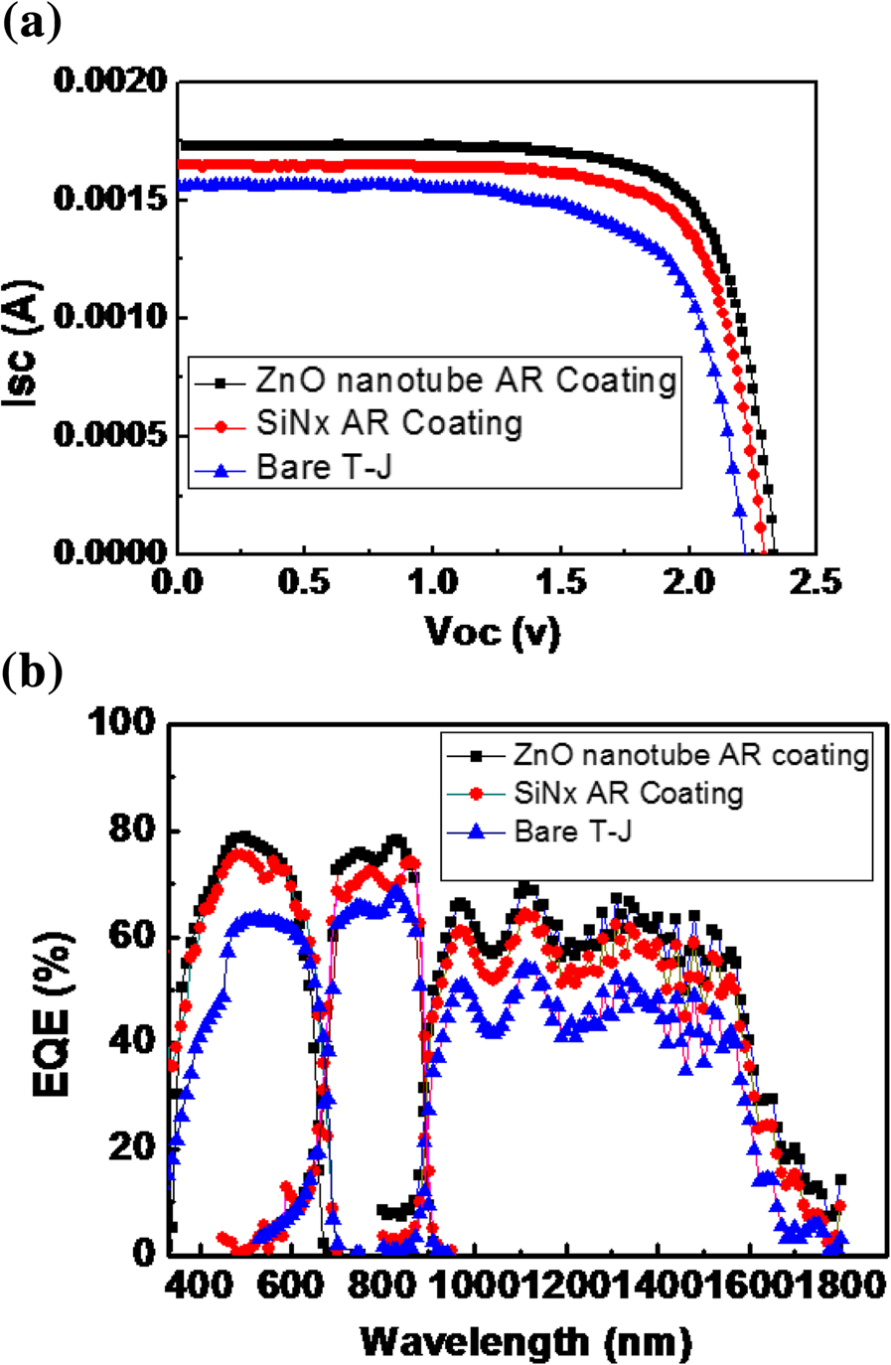
**I-V characteristics of T-J solar cells and External quantum efficiency. (a)** Photovoltaic I-V characteristics of T-J solar cell with and without Si_3_N_4_and ZnO nanotube structure, respectively. **(b)** External quantum efficiency of bare triple-junction (T-J) solar cell and T-J solar cell with SiN_4_ and ZnO nanotube coating, respectively.

The photovoltaic I-V characteristics were measured under one sun AM1.5 (100 mW/cm^2^) solar simulator. The device parameters of open-circuit voltage (*V*_oc_), short-circuit current (*I*_sc_), fill factor (FF) conversion efficiency (*η*), and quantum efficiency (QE) were measured. Figure 5a shows the I-V characteristics of T-J solar cells with and without a Si_3_N_4_ and ZnO nanotube structure. The efficiencies of a T-J solar cell with and without a Si_3_N_4_ and ZnO nanotube structure are 19.3, 22.5, and 24.2%, respectively, as shown in Table 1. The short-circuit current density (*J*_sc_) increased from 12.5 to 13.2 and 13.2 to 13.9 mA/cm^2^ after the addition of a Si_3_N_4_ and ZnO nanotube on the solar cell, and the *J*_sc_ was improved 5.3% in enhancement in overall power conversion efficiency. The largest efficiency and *J*_sc_ values were obtained for the T-J solar cell with ZnO nanotube. The reason for this is that a ZnO nanotube decreases the reflectance and increases the short-circuit current. The quantum efficiency of a solar cell is defined by the following equation:

**Table 1:** **Measured illuminated electrical properties of bare triple (T-J) solar cell and T-J solar cell with SiN**_**4 **_**and ZnO nanotube coating, respectively**

**Sample**	***V***_**oc **_**(V)**	***J***_**sc**_**(mA/cm**^**2**^**)**	**FF (%)**	**Efficiency (%)**
Bare T-J solar cell	2.2	12.5	71.2	19.3
With SiNx AR coating	2.3	13.2	74.5	22.5
With ZnO NW AR coating	2.3	13.9	74.8	24.2

(1) $$\mathrm{QE}=J_{\mathrm{sc}}\left(\lambda \right)\mathrm{hc}/\mathrm{q\phi}\left(\lambda \right)$$

where *J*_*sc*_ (*λ*) is the total photogenerated short-circuit current density at a given wavelength *λ*, *ϕ*(*λ*) is the photon flux of the corresponding incident light, and *q* is the elementary charge [18]. We measured the spectral response of the external quantum efficiency (EQE), in which a xenon lamp and a halogens lamp were used as the illumination source sources. The EQE of the T-J solar cell device with SiN4 and ZnO nanotube coating, respectively, are presented in Figure 5b. Physically, EQE means the ability to generate electron-hole pairs caused by the incident photon [19]. The cell with ZnO nanotube coating shows an enhanced EQE in a range from of 350 to 1800 nm. The average EQE enhancements (△EQE) of the top and middle cells were 2.5 and 6.6%, respectively. This is due to the low reflection between the wavelength 350 to 500 nm, in respect to the solar cell coated with a ZnO nanotube. The photocurrent generated in cell is in proportion to values of EQE and will optimize the III-V solar cell. The T-J solar cell is built by three series subcells, in which each subcell provides a short circuit current (*J*_sc_ 1, *J*_sc_ 2, *J*_sc_ 3) and open circuit voltage (*V*_oc_ 1, *V*_oc_ 2, *V*_oc_ 3). The total *V*_oc_ is the sum of three subcells and *J*_sc_ is limited by the smallest one. The short circuit limits of the current density of the top and middle cell can be calculated by ref. [20].

## Conclusions

A ZnO nanotube grown on triple-junction (T-J) solar cell devices by the hydrothermal growth method to enhance efficiency is investigated. The reflectance spectra and I-V characteristics indicate that the ZnO nanotube solar cell had the lowest reflectance, especially in the range of 350 to 500 nm from ultraviolet to visible light. Solar cells with a ZnO nanotube exhibited a conversion efficiency increase of 4.9% compared with a bare T-J solar cell, whereas T-J solar cells with SiNx AR coating had only a 3.2% increase. After encapsulation, the results also suggested that the cell with ZnO nanotube coating could provide the best solar cell performances.

## Competing interests

The authors declare that they have no competing interests.

## Authors' contributions

CCC, BTT, and KLL carried out the InGaP/GaAs/Ge solar cell process and hydrothermal growth of ZnO nanotube and drafted the manuscript. YTH and HWY carried out the device measurements, including I-V, QE, and reflectance. NHQ carried out material analysis, including TEM and SEM. EYC conceived this work and participated in its design and coordination. All authors read and approved the final manuscript.
